# Evaluation of a Digital Clinician-Training Module for Treatment of Transgender and Gender Diverse Patients With Eating Disorders: Pre-Post Study

**DOI:** 10.2196/84937

**Published:** 2026-09-03

**Authors:** Nora Yanyi Sun, Alex S Keuroghlian

**Affiliations:** 1 Department of Statistics, Harvard University, 373 Quincy Mail Center, Cambridge, MA, 02138, United States, 1 9046468255; 2 Department of Behavioral Health, MaineHealth, Portland, ME, United States

**Keywords:** eating disorders, disordered eating, continuing medical education, health education, LBTQIA+, transgender, gender diverse

## Abstract

A digital clinician training module significantly improved attitudes, beliefs, and self-perceived competence in a cohort of medical students (n=87) toward working with transgender and gender diverse (TGD) people with eating disorders. Future clinician training resources can adopt this cost-effective and accessible learning model and build upon existing introductory attitude- and awareness-based programs by focusing on addressing a specific health disparity among TGD people.

## Introduction

Among transgender and gender diverse (TGD) people, 18% were diagnosed with an eating disorder (ED) in the past year, compared to <2% of cisgender people [1]. TGD people experience internal stressors, such as gender dysphoria, and external stressors, such as discrimination, which contribute to an elevated rate of EDs.

Clinicians may receive insufficient medical training about EDs [2]. Few clinicians are specifically educated on how to care for TGD people with EDs, and thus they may fail to address the unique challenges faced by these patients, hindering their recovery [3]. Furthermore, clinicians report variable attitudes toward working with TGD patients, which may be improved through convenient and accessible targeted digital training [4]. However, no in-depth clinician training activities currently focus on TGD people with EDs [5].

In this study, we report on the development and evaluation of a training module on treating TGD people with EDs for health care workers in primary care settings (Multimedia Appendix 1). The structure of the module aligns with principles of adult learning theory (eg, andragogy) and student-centered learning (eg, scaffolding) by emphasizing self-directed, practice-based learning and application of progressively more advanced skills to clinical scenarios [6].

As lack of institutional support represents a significant obstacle to producing clinician-training materials for marginalized populations, we demonstrate the feasibility of a digital approach requiring minimal technical and financial support [6].

## Methods

### Module Structure

Developed by the authors, the module is intended to be completed independently in approximately one hour (Multimedia Appendix 1). The learner advances through 6 sections in the slide deck, including an overview, instructional slides, and ungraded, interactive knowledge checks, where learners can click the question slide to reveal the answer. The module is hosted on Google Slides, allowing the learner to skip or navigate sections in any order.

### Module Evaluation

Participants were Harvard Medical School medical students recruited using a school-wide mailing list. Students completed a preintervention survey, the module, and a postintervention survey via email.

In the preintervention and postintervention surveys, participants rated their agreement with a set of statements designed based on prior studies [7-9] that evaluated beliefs (preintervention: α=.87; postintervention: α=.88), attitudes (preintervention: α=.88; postintervention: α=.93), and self-efficacy (preintervention: α=.87; postintervention: α=.85) toward working with TGD patients with EDs on a 7-point Likert-scale from “strongly disagree” (1) to “strongly agree” (7). The survey items are shown in the Results section. Sociodemographic details and participant satisfaction were also collected [7,10]. Participants were required to complete all questions.

Using R (version 4.4.1; R Project for Statistical Computing) in Rstudio, mean and SD for the survey data were calculated; mean differences and the significance level between paired questions were determined with paired *t* tests, a method that is robust for n>30.

### Ethical Considerations

The Harvard-Longwood Campus Institutional Review Board approved this study (IRB24-0636). Informed consent was collected. All data were deidentified, and participants received a US $50 e-gift card.

## Results

Of 102 participants who completed the preintervention survey, 87 (85%) also completed the postintervention survey and were included in the study. Most participants were cisgender, white or Asian, and had TGD and/or lesbian, gay, bisexual, transgender, queer/questioning, intersex, and asexual (LGBTQIA+) friends (Table 1).

**Table 1.** Demographic characteristics of Harvard Medical School students who completed the digital clinician training module for treatment of transgender and gender diverse patients with eating disorders in this pre-post study (n=87).

Characteristics	Values
Age (years), mean (SD)	25.35 (1.9)
Gender identity, n (%)	
Man	30 (34.5)
Woman	49 (56.3)
Nonbinary	6 (6.9)
Genderqueer or not exclusively female or male	2 (2.3)
Gender modality, n (%)	
Cisgender	83 (95.4)
Transgender	4 (4.6)
Sexual orientation, n (%)	
Straight or heterosexual	36 (41.4)
Gay	12 (13.8)
Lesbian	3 (3.5)
Bisexual	18 (20.7)
Pansexual	2 (2.3)
Queer	12 (13.8)
Asexual	3 (3.5)
Questioning	1 (1.2)
Race, n (%)	
American Indian/Alaskan Native	0 (0)
Asian	30 (34.5)
Black/African American	2 (2.3)
Native Hawaiian/Pacific Islander	0 (0)
White	43 (49.4)
Something else	3 (3.5)
Multiracial	9 (10.3)
Ethnicity, n (%)	
Hispanic or Latina/e/o/x	7 (8.1)
Not Hispanic or Latina/e/o/x	80 (92.0)
Have transgender family or friends, n (%)	
Yes	63 (72.4)
No	24 (27.6)
Have LGBTQIA+^a^ family or friends, n (%)	
Yes	82 (94.3)
No	5 (5.8)
Year of medical school, n (%)	
MS^b^1	14 (16.1)
MS2	33 (37.9)
MS3	18 (20.7)
MS4	22 (25.3)

a LGBTQIA+: lesbian, gay, bisexual, transgender, queer, intersex, asexual, and all sexually and gender diverse.

b MS: medical school.

As shown in Table 2, paired 2-tailed *t* tests identified significant increases in all 3 subsections; the largest increases were observed on self-efficacy statements, such as identifying harm reduction strategies for gender dysphoria (+2.39; *P*<.001). Only 2 statements, on believing TGD identities are valid and being interested in learning about TGD health interventions, did not significantly increase, potentially due to high pretest scores. Participants indicated high levels of satisfaction, with mean ratings between 5.70 and 6.45.

The module was improved based on feedback (eg, adding more case study questions); the module will be made eligible for continuing medical education credits and promoted through the National LGBTQIA+ Health Education Center.

**Table 2.** Pre- versus postintervention beliefs, attitudes, and self-perceived competence and postintervention satisfaction among Harvard Medical School students who completed the digital clinician training module for treatment of transgender and gender diverse patients with eating disorders in this pre-post study. Agreement with each statement was rated agreement on a 7-point Likert scale from “strongly disagree” (1) to “strongly agree” (7).

Items	Preintervention rating, mean (SD)	Postintervention rating, mean (SD)	Mean difference	*P* value
Beliefs				
I believe that a person’s gender can differ from their biological sex assigned at birth.	6.60 (1.15)	6.75 (0.89)	0.15	.06
I believe that affirming a transgender and gender diverse patient’s gender identity is important for their health.	6.61 (1.04)	6.72 (0.96)	0.11	.01
I believe that insurance should cover gender-affirming medical interventions, such as gender-affirming surgeries or gender-affirming hormone therapy.	6.38 (1.35)	6.53 (1.27)	0.15	.004
I believe that transgender and gender diverse patients with eating disorders face unique obstacles to recovery.	6.45 (0.92)	6.78 (0.65)	0.33	<.001
Attitudes				
I am personally interested in learning more about health interventions for transgender and gender diverse patients.	6.18 (1.29)	6.31 (1.27)	0.13	.09
I believe that my medical school curriculum should include more training about caring for transgender and gender diverse patients.	6.10 (1.29)	6.28 (1.33)	0.17	.02
Self-perceived competence				
I understand non-cisgender gender identities, and the difference between gender and sex.	6.44 (0.96)	6.80 (0.50)	0.37	<.001
I can use culturally responsive, gender-affirming language with transgender and gender diverse patients.	5.98 (0.94)	6.60 (0.56)	0.62	<.001
I can screen for common eating disorders, such as anorexia nervosa, bulimia nervosa, and binge-eating disorder.	4.41 (1.60)	5.83 (1.09)	1.41	<.001
I can identify symptoms of gender dysphoria among eating disorder patients.	4.00 (1.54)	5.85 (1.07)	1.85	<.001
I understand how gender dysphoria may result in disordered eating behaviors among transgender and gender diverse patients.	4.67 (1.64)	6.31 (0.80)	1.64	<.001
I can identify unique treatment obstacles (aside from gender dysphoria) that transgender and gender diverse patients recovering from eating disorders may face.	4.29 (1.58)	6.13 (0.83)	1.84	<.001
I can identify harm reduction strategies that minimize gender dysphoria for transgender and gender diverse patients whose eating disorders may be influenced by gender dysphoria.	3.60 (1.56)	5.99 (0.92)	2.39	<.001
I can assess and manage common co-occurring physical and mental health problems among transgender and gender diverse patients with eating disorders.	3.60 (1.64)	5.39 (1.27)	1.79	<.001
Satisfaction				
The module provided new ideas or information, enhancing my current knowledge base.	—^a^	6.45 (0.92)	—	—
Division of the content into six sections furthered my understanding of the content.	—	6.14 (1.02)	—	—
The informational presentation slides furthered my understanding of the content.	—	6.38 (0.77)	—	—
The case study questions furthered my understanding of the content.	—	6.37 (0.97)	—	—
The multiple-choice questions furthered my understanding of the content.	—	6.20 (1.07)	—	—
The short response questions furthered my understanding of the content.	—	5.70 (1.29)	—	—
I am able to apply the information presented if I were to treat transgender and gender diverse patients with eating disorders.	—	6.00 (1.00)	—	–

a Not applicable.

## Discussion

To address disparities in ED risk and outcomes among TGD patients, we developed an hour-long self-paced learning module that included 6 sections with accompanying review questions after each section, and we piloted the module with a cohort of 87 medical school students. Prior to completing the module, students scored highly in the attitudes and beliefs subsections but lower in the self-efficacy subsection, suggesting that while the vast majority of students supported TGD people and had TGD and/or LGBTQIA+ friends, they remained uncertain about how to help TGD patients in a clinical setting. After completing the education module, the students showed statistically significant improvements in all 3 subsections, with the largest improvements in self-efficacy. Students reflected that they gained valuable information from the module, oftentimes despite already having some familiarity with the topic, and they felt confident applying this new information in a clinical setting.

Consistent with prior studies and adult learning theory [6], students found application-based questions to be most helpful. In particular, students reported that the case study questions, which present hypothetical patient care scenarios, meaningfully improved their self-efficacy toward applying concepts in the clinical setting.

Results are limited by the student participant pool, who may not be representative of primary care workers, especially those from different locations and/or political climates. Social desirability bias may have skewed the scores, and skills-based learning was not tested. Participant interviews may further elucidate module strengths and weaknesses; the module may also be enhanced through interactive elements not possible in Google Slides, such as clickable answers.

Supportive and respectful attitudes alone are insufficient to ensure that TGD patients receive high-quality medical care. During a time of limited institutional support for LGBTQIA+ health equity, publicly and digitally accessible clinician training activities that apply this free, simple, and effective model can allow students to learn more advanced clinical skills for addressing a specific health disparity in a subpopulation of LGBTQIA+ patients.

## Supplementary material

10.2196/84937 Multimedia Appendix 1 Digital clinician training module.

## Abbreviations

ED: eating disorder
LGBTQIA+: lesbian, gay, bisexual, transgender, queer/questioning, intersex, and asexual
TGD: trans and gender diverse
